# Site- and structure-specific characterization of glycoproteins of H11: potent vaccine candidates against parasitic worm *Haemonchus*

**DOI:** 10.3389/fimmu.2025.1669536

**Published:** 2026-01-05

**Authors:** Xin Liu, Feng Liu, Hui Liu, Lisha Ye, Yao Zhang, Wenjie Peng, Nishith Gupta, Min Hu, Chunqun Wang

**Affiliations:** 1 National Key Laboratory of Agricultural Microbiology, College of Veterinary Medicine, Huazhong Agricultural University, Wuhan, China; 2 Shanghai Center for Systems Biomedicine, Shanghai Jiao Tong University, Shanghai, China; 3 Intracellular Parasite Education and Research Labs (iPEARL), Department of Biological Sciences, Birla Institute of Technology and Science, Pilani (BITS Pilani), Hyderabad, India

**Keywords:** parasitic worm, N-glycoproteome, intact N-glycopeptide analysis, vaccine, glycan analysis

## Abstract

Parasitic worm (helminth) infections pose significant threats to global health and livestock productivity. Although mass spectrometry (MS)-based glycomics have revealed that helminths express structurally complex N-glycans, site- and structure-specific characterization of intact glycopeptides remains a major challenge. Here we employed advanced MS-based intact glycoproteomics to explore the N-glycosylation profile of H11 antigen – an important vaccine antigen derived from the pathogenic parasite *Haemonchus contortus*. We identified seven glycosylated aminopeptidases carrying 19 N-glycosylation sites with 31 distinct N-glycan structures. Notably, 13 N-glycopeptides were significantly enriched by H11-induced protective IgG antibodies. Our results revealed extensive structural heterogeneity and abundant core fucosylation among the identified N-glycopeptides. Additionally, molecular docking analyses demonstrated that these IgG-recognized N-glycopeptides were located on the protein surface or near substrate-access channels, indicating their potential as antigenic epitopes. Overall, this work provides a precise glycoproteomic characterization of a key helminth antigen and offers valuable insights for the rational design of vaccines against *H. contortus* and other related parasitic species.

## 1 Introduction

Asparagine (N)-linked glycosylation of proteins is one of the most prevalent and functionally significant post-translational modifications (PTMs) in eukaryotic organisms. It plays essential roles in protein folding, intracellular trafficking, receptor-ligand recognition, immune regulation, and disease progression (1, 2). This PTM exhibits substantial structural diversity, as glycans with various compositions can be covalently attached to multiple asparagine residues within a single polypeptide (3, 4). Mass spectrometry (MS) has emerged as the foundation of glycomics and glycoproteomics research. However, conventional MS-based approaches often depend on enzymatic deglycosylation. Although this facilitates the characterization of glycan structures, it eliminates valuable site-specific information regarding the glycoprotein heterogeneity (5, 6). Consequently, it is still challenging to determine whether certain glycosylation sites have a preferential association with specific glycan structures. In recent years, advanced MS-based intact glycoproteomics approaches have been developed to preserve both the peptide backbone and the attached glycan moieties, thereby enabling comprehensive mapping of site-specific glycosylation (7). Nevertheless, these powerful tools remain underutilized across many biological systems and organisms.

Parasitic worms (helminths) are a major global health and agricultural burden, causing chronic infections in both humans and animals. Current control strategies rely primarily on anthelmintic drugs; however, extensive and often indiscriminate usage has resulted in the emergence of widespread drug resistance, particularly among gastrointestinal nematodes (8–10). Despite substantial efforts to develop vaccines against helminth infections, most candidates—whether native or recombinant—have failed to achieve consistent and robust protection (11, 12). Notably, accumulating evidence indicates that native helminth glycoproteins play crucial roles in eliciting host immune responses, with their glycosylation patterns potentially modulating antigenicity and vaccine efficacy (13–15).

H11 is a well-characterized glycoprotein complex derived from *Haemonchus contortus* – a highly pathogenic gastrointestinal nematode in livestock (16). This antigen has demonstrated high protective efficacy (75–95%) in field trials and has been extensively studied as a vaccine candidate against haemonchosis (17). H11 consists primarily of aminopeptidases located on the microvillar surface of the parasite’s intestinal epithelium, where they play an essential role in nutrient digestion and parasite survival (18, 19). Our recent findings in glycomics and glycoproteomics analyses of H11 demonstrate that protective immunity is closely associated with the N-linked glycan structures of H11 glycoproteins (15). However, the comprehensive characterization of how diverse glycans are linked and distributed across multiple glycosylation sites remains poorly understood.

Given the structural heterogeneity of glycoproteins, this study employed advanced MS-based intact glycoproteomic approaches to perform a comprehensive site-specific glycosylation analysis of the H11 antigen. The findings highlight the importance of detailed glycoproteomic profiling in understanding parasite biology and provide new insights to inform the rational design of vaccines targeting metazoan parasites.

## 2 Materials and methods

### 2.1 Biological reagents and chemicals

Con-A Sepharose 4B was obtained from GE Healthcare (Pittsburgh, PA, USA). Acetonitrile (ACN), dithiothreitol (DTT), trifluoroacetic acid (TFA), Tris (2-carboxyethyl) phosphine (TCEP), iodoacetamide (IAA), ammonium bicarbonate (NH_4_HCO_3_) and trypsin NBr™ sepharose 4B were purchased from Sigma Aldrich (St. Louis, MO, USA). Ammonium bicarbonate (NH_4_HCO_3_) and bicinchoninic acid (BCA) protein assay kit were purchased from Sangon Biotech (Shanghai, China). Ultrapure water was produced using Millipore Simplicity System (Billerica, MA, USA).

### 2.2 Sample preparation of H11 antigen

Native H11 antigen was isolated from adult *H.* contortus (Haecon-5 strain) using ConA-sepharose affinity chromatography as previously described (12, 20). Briefly, 15 g of adult worms were homogenized in ice-cold phosphate-buffered saline (PBS, pH 7.4) for 25 min using a glass homogenizer. The homogenate was centrifuged at 12,000 g for 25 min, and the resulting pellets were extracted 4 times with 1% (v/v) Thesit in PBS, followed by filtration through a 0.45 μm membrane. The extract was loaded on a ConA-Sepharose column, which was washed 3 times with 20 mM Tris-HCl buffer, and subsequently eluted with a 200 mM solution of methyl α-D-mannopyranoside and methyl α-D-glucopyranoside. Fractions containing H11 were pooled and filtered through a 0.22 μm membrane. The resulting filtrate was designated as native H11 antigen. The protein concentration was determined using a BCA assay kit.

### 2.3 Anti-H11 IgG antibodies

The IgG antibodies used in this study were derived from our previous study (15). Briefly, anti-NA IgG was purified by Protein A+G agarose from the sera of goats immunized with natural H11 antigen (NA group). In contrast, anti-PI IgG was obtained from the sera of goats immunized with deglycosylated H11 antigen, which had been treated with sodium periodate to destroy glycans.

### 2.4 Sample preparation of intact N−glycopeptides

**Figure 1:**
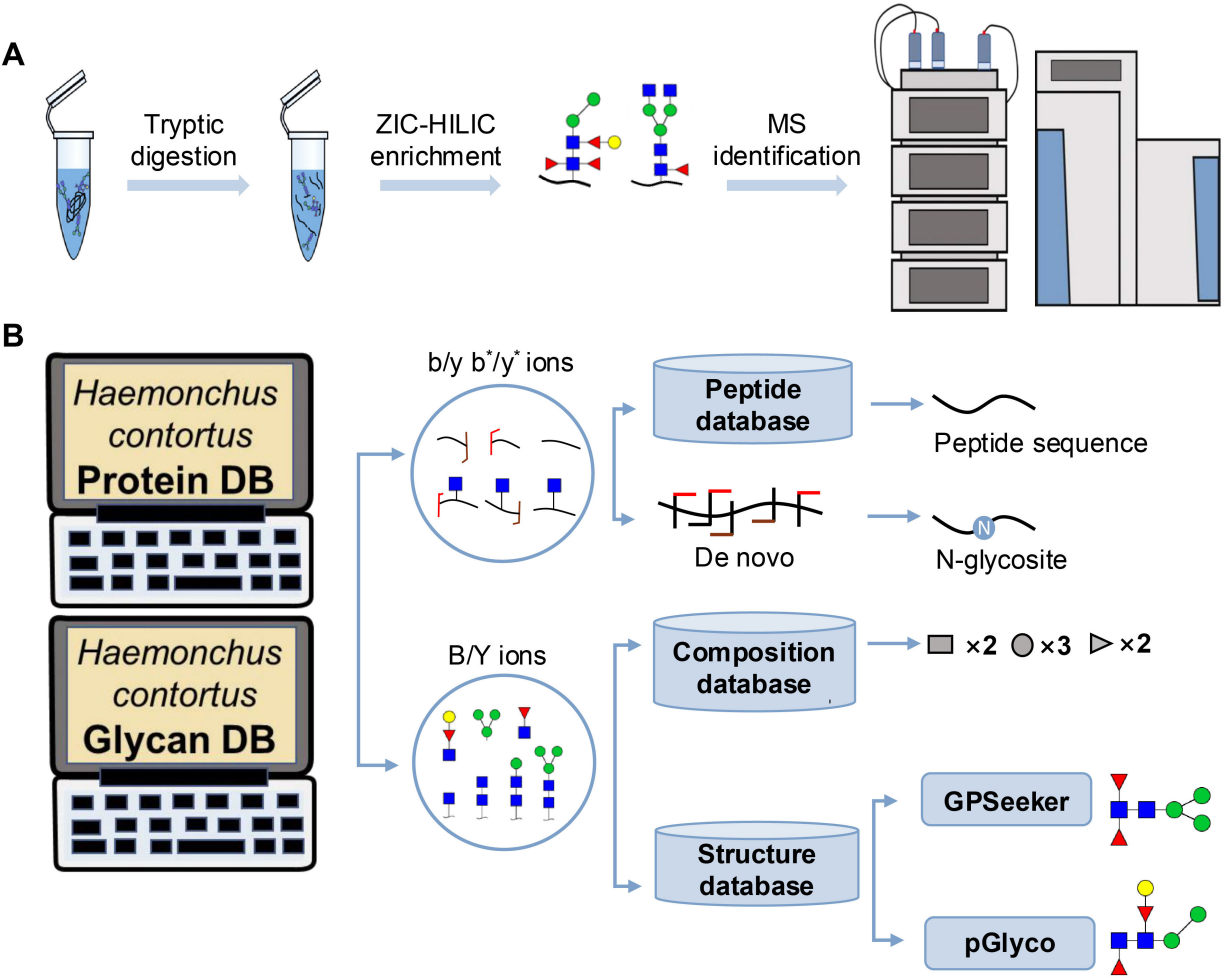
Workflow of mass spectrometry (MS)-based intact glycoproteome analysis of H11 antigen. **(A)** H11 glycoproteins were subjected to trypsin digestion, ZIC-HILIC enrichment and MS identification. **(B)** Comprehensive N-glycoproteome databases were based on known H11 glycome and protein isoforms (15, 22). Data analysis was performed using b/y product ions to determine the peptide backbone at GlcNAc-containing sites, while GPSeeker and pGlyco were used to map the structure-diagnostic B/Y product ions from the N-glycan structures. Glycans were annotated using the symbol nomenclature (green circle, mannose; yellow circle, galactose; blue square, GlcNAc; red triangle, fucose).

The workflow for intact N-glycopeptide analysis is illustrated in **Figure 1**. Briefly, ConA-purified H11 glycoprotein was reduced with 10 mM DTT at 55 °C for 30 min, alkylated with 20 mM IAA at room temperature in the dark for 20 min, and quenched with an additional 10 mM DTT. The reduced and alkylated protein was dissolved in 5 mL of 50 mM NH_4_HCO_3_ and digested with 20 μg of trypsin at 37 ˚C overnight. The resulting peptides were desalted using homemade C18 solid-phase extraction (SPE) tips (Jupiter C18, 15 μm particle size, 300 Å pore size). The C18 tips were preconditioned sequentially with 80% ACN containing 5% TFA and then with 0.1% TFA. The digested peptides were loaded onto the C18 tips, washed with 0.1% aqueous TFA, and eluted with 100 μL of 50% ACN containing 0.1% TFA followed by 100 μL of 80% ACN containing 5% TFA. The eluates were dried in a SpeedVac and reconstituted in 100 μL of 80% ACN containing 5% TFA. Intact N-glycopeptides were enriched using homemade SPE columns packed with ZIC-HILIC particles (Merck Millipore, 5 μm particle size, 200 Å pore size). The columns were equilibrated with 0.1% aqueous TFA and then followed by 80% ACN containing 5% TFA. The peptide mixtures were loaded, washed with 80% ACN containing 5% TFA (21), and eluted with 100 μL of 0.1% aqueous TFA and with 50 μL of 50 mM NH_4_HCO_3_. The combined eluates were dried in a SpeedVac and resuspended in 40 μL of water for subsequent MS/MS analysis.

### 2.5 Nano-RPLC ESI-MS/MS analysis

Samples were analyzed using a Dionex Ultimate 3000 RSLCnano (Thermo Fisher Scientific) coupled to an Orbitrap Exploris 480 (Thermo Fisher Scientific) operating in the positive ion mode. Chromatographic separation was performed on a C18 analytical column (ZORBAX 300SB, 5 μm, 300 Å; Agilent Technologies, Santa Clara, CA, USA) with dimensions of 60 cm × 360 μm (o.d.) × 75 μm (i.d.). The separation was carried out at a flow rate of 300 nL/min using mobile phases consisting of (A) 99.9% H_2_O with 0.1% FA and (B) 99.9% ACN with 0.1% FA. The LC gradient was programmed as follows: 2% B for 12 min; 2–40% B over 180 min; 40–95% B over 5 min; 95% B for 5 min; 95–2% B over 5 min; and 2% B for 20 min for column re-equilibration. Electrospray ionization (ESI) parameters were set as follows: spray voltage, 1.9 kV; capillary temperature, 300 °C. Full-scan MS spectra were acquired over an *m/z* range of 700–2000 with a resolution of 60,000 (at *m/z* 200), an automatic gain control (AGC) target of 3 × 10^6^, and a maximum ion injection time of 20 ms. MS/MS spectra were acquired on Top20 data-dependent acquisition mode with the following settings: mass resolution 30,000, AGC target, 5 × 10^5^, maximum injection time, 250 ms, isolation window, 3 m/z, and stepped higher-energy collisional dissociation (HCD) with normalized collision energies of 20%, 30% and 40%. Dynamic exclusion was set to 20 s.

### 2.6 Database search and identification of intact N-glycopeptides

All intact N-glycopeptides included in the database were constructed in silico by exhaustively combining the computationally predicted N-glycosite-containing tryptic peptides with 50 experimentally identified *H. contortus* N-glycan structures (**Supplementary Table 1**) (15). The N-glycosite-containing peptides were derived from *H. contortus* aminopeptidase protein sequences obtained from UniProt (https://www.uniprot.org/) using the organism filter “*H. contortus* aminopeptidase,” resulting in the selection of 13 known aminopeptidases (**Supplementary Table 2**) (22). The intact N-glycopeptide database was divided into two discrete subsets to enable parallel interrogation through two specialized search engines, employed independently for separate analyses as described in a previous study (23). Briefly, GPSeeker was performed to analyze glycan structures containing a core pentasaccharide, whereas pGlyco complements this capability by covering glycan sequences that lack the core pentasaccharide. The intact N-glycopeptide database was divided into two discrete subsets and analyzed independently by two complementary search engines. GPSeeker was employed to identify glycopeptides containing a conserved N-glycan pentasaccharide core, whereas pGlyco 3.0 complemented this by detecting glycopeptides lacking the core pentasaccharide structure.

Each raw MS dataset was analyzed separately using GPSeeker and pGlyco 3.0 to generate two sets of intact N-glycopeptide identifications (IDs) with a false discovery rate (FDR) < 0.01. Each ID set was refined by removing redundant entries and filtering until no MS² spectrum was assigned to multiple N-glycopeptide matches within each engine’s output. The remaining IDs from both search engines were then merged to produce an engine-union qualitative ID list. It is noteworthy that a small subset of MS² spectra was independently assigned to different N-glycopeptides by the two engines due to the presence of structural isomers in the split databases. To ensure a unique N-glycopeptide assignment for each MS² spectrum, the identification exhibiting the greater number of matched peptide and glycan fragment ions was retained as the final interpretation.

### 2.7 Silver staining analysis of IgG-recognized H11 proteins

Anti-NA or anti-PI IgG antibodies (50 μg/mL, 1 mL) were incubated with Protein A+G beads at room temperature for 30 min. Subsequently, native H11 samples were added to the antibody–bead mixture and incubated at 4 °C for 3 h. The magnetic beads were separated using a magnetic stand and washed with TBS. Subsequently, resuspended in the sample loading buffer and boiled for 10 min. After brief magnetic separation (10 s), the supernatant was collected and subjected to silver staining analysis.

### 2.8 Identification of IgG-bound H11 antigen

The workflow was performed according to a modified protocol (24). Briefly, 250 μg of anti-NA or anti-PI IgG were each dissolved in 100 mM NaHCO_3_ buffer (pH 8.5) containing 500 mM NaCl. In parallel, 0.5 mL of CNBr-activated Sepharose beads were swollen in 1 mM HCl at 4 °C for 30 min, washed with 10 mL ddH_2_O to remove residual acid, and subsequently equilibrated with 1 mL of NaCl/NaHCO_3_ buffer (pH 8.5) to activate the resin. The activated beads were immediately transferred into the corresponding IgG-containing coupling solution. After incubation at room temperature for 2 h, the mixture was centrifuged at 13,000 × *g* for 1 min, and the supernatant was discarded. The agarose beads were then washed with 2 mL of NaHCO_3_/NaCl buffer to remove unbound antibodies.

The H11 glycoproteins were first incubated with the anti-PI IgG–coupled beads at room temperature for 4 h. The unbound fraction (supernatant) was then collected and subsequently incubated with the anti-NA IgG–coupled beads under the same conditions for 4 h. After centrifugation, the pellets were thoroughly washed four times with 25 mM ammonium acetate (pH 7.5) to remove non-specifically bound proteins. The NA-IgG–bound proteins were eluted by incubation with 1 mL of 25 mM ammonium acetate (pH 3.0) for 30 min. Finally, the eluted fractions were subjected to MS-based intact glycopeptide analysis following the established procedures described in Sections 2.4 (*Sample preparation of intact N-glycopeptides*) and 2.5 (*Nano-RPLC ESI-MS/MS analysis*).

### 2.9 Analysis of intact N-glycopeptides

Quantitative analysis of each N-glycopeptide was performed by summing the intensities of the isotopic peaks corresponding to the intact N-glycopeptide precursors. IDs with a lack of the precursor top peak matches were eliminated from the processes of quantitative analysis. The relative abundance of each quantitative N-glycopeptide ID was determined using diverse normalization methods, including sum normalization, max-min normalization, mean normalization, Z-score normalization, and log normalization. The quantitative identification was constructed by selecting consistently identified N-glycopeptides, allowing for missing identifications in up to 50% of the samples within the same group. Missing values were imputed using the neighboring value multiplied by a normalization factor. The normalization factor was determined by first filtering a set of inter-sample shared N-glycopeptides exhibiting extremely high quantitative linear correlation, followed by calculating the ratio between samples.

### 2.10 Molecular docking

The tertiary structure of the H11 protein PDB files was obtained from the AlphaFold Protein Structure Database (https://alphafold.ebi.ac.uk/); SDF files of the small molecule Leu-pNA (CNS No. 4178-93-2) were downloaded from the PubChem database and converted to PDB files using PyMOL. After hydrogenation, charge calculation, merging non-polar hydrogen and other operations, the generated structure was saved as a pdbqt file. The algorithm was executed to optimize local-search parameters with more than 50 independent runs; resulting docking poses were ranked by predicted binding energy and the compound (pose) with the lowest binding energy was selected as the top candidate. Autodock programme was used for docking, while PyMOL was used to visualize the docking data and the amino acid residues that form hydrogen bonds in the 4 Å range. Surface accessibility and position distribution analyses were carried out using the NetSurfP server (https://services.healthtech.dtu.dk/services/NetSurfP-3.0/). N-glycopeptide enrichment analyses were performed via the Hiplot platform (https://hiplot.com.cn/home/index.html/) and the Microbiotics website (http://www.bioinformatics.com.cn/).

## 3 Results

### 3.1 Experimental design

We previously analyzed the N-glycome and N-glycoproteome profile of *H. contortus* (15, 25); however, the precise correlation between specific glycosylation sites and their corresponding glycan structures has remained unresolved. To address this gap, we conducted a comprehensive site- and structure-specific characterization of N-glycosylation within H11 components. An integrated N-glycoproteomics workflow was employed, combining tryptic digestion, ZIC-HILIC enrichment, and MS identification (**Figure 1**, **Supplementary Tables 1, 2**). For MS spectral analysis, b/y ions were used to identify GlcNAc-carrying peptide fragments, and the GPSeeker and pGlyco 3.0 algorithms were applied to characterize glycan compositions via B/Y ion mapping (**Figure 1B**), a strategy well established in N-glycoproteome analysis (26).

### 3.2 Identification of intact N-glycopeptides of H11 antigen

**Figure 2:**
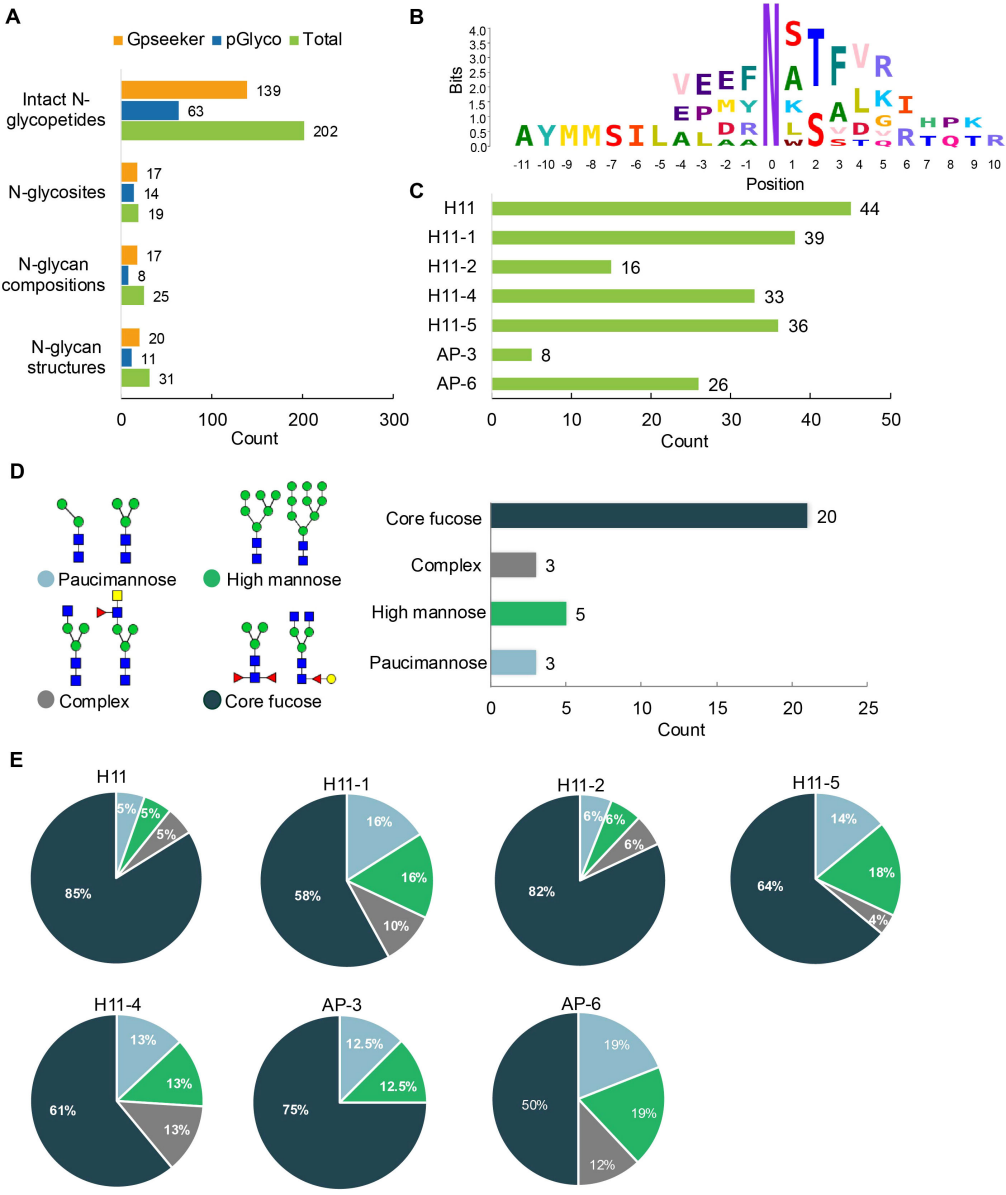
Characteristics of the intact N-glycopeptide profile of H11 antigen. **(A)** Information on the numbers of intact N-glycopeptides, N-glycosites, N-glycan compositions and N-glycan structures obtained from GPSeeker and pGlyco techniques, respectively. Data were derived from two independent experiments. **(B)** Motif analysis of all identified N-glycopeptides. **(C)** Relative percentage of intact N-glycopeptides identified in each H11 aminopeptidase. **(D)** The representative glycan structures and the percentage distribution of N-glycan classes. Glycans were annotated using the symbol nomenclature (green circle, mannose; yellow circle, galactose; blue square, GlcNAc; yellow square, GalNAc; red triangle, fucose). **(E)** Percentages of the four types of N-glycans identified in each H11 aminopeptidase.

A total of 202 intact N-glycopeptides were identified, corresponding to 19 N-glycosylation sites (**Figure 2A**, **Supplementary Table 3**). Conserved N-x-T/S sequons (x ≠ proline) exhibited a pronounced preference for threonine over serine (**Figure 2B**), consistent with our previous observation in *H. contortus* (15, 24). Isoform-specific analysis showed that H11 antigen comprised five known isoforms (H11, H11–1, H11–2, H11–4 and H11–5) (12, 27–29) and two aminopeptidase molecules (AP-3 and AP-6) (22), with H11, H11-1, H11-4, H11-5 and AP-6 showing predominant expression (**Figure 2C**).

Glycomic profiling identified 25 distinct N-glycan compositions corresponding to 31 structures (**Figure 2A**, **Supplementary Table 3**), which were classified into four types, i.e., paucimannose (Hex_2-4_HexNAc_2_), high-mannose (Hex_5-9_HexNAc_2_), complex (Hex_3-4_HexNAc_3-5_Fuc_0-2_) with or without core fucose (Hex_2-4_HexNAc_2_Fuc_0-3_) (**Figure 2D**). Notably, 65% (n=20) of the identified structures exhibited α1,3- and/or α1,6-fucosylation (**Figure 2D**, **Supplementary Table 3**). In H11, H11–2 and AP-3, over 75% structures are core fucosylated (**Figure 2E**). Core fucosylation has been demonstrated to act as a pathogenic and immunological glyco-determinant in helminth N-glycans (15). Accordingly, our intact N-glycopeptide analysis deepens the understanding that ‘non-mammalian’ glycans, including α1,3-core fucose, may serve as critical elements for H11 antigen in stimulating host immune response.

### 3.3 Heterogeneity analysis of H11 glycoproteins

**Figure 3:**
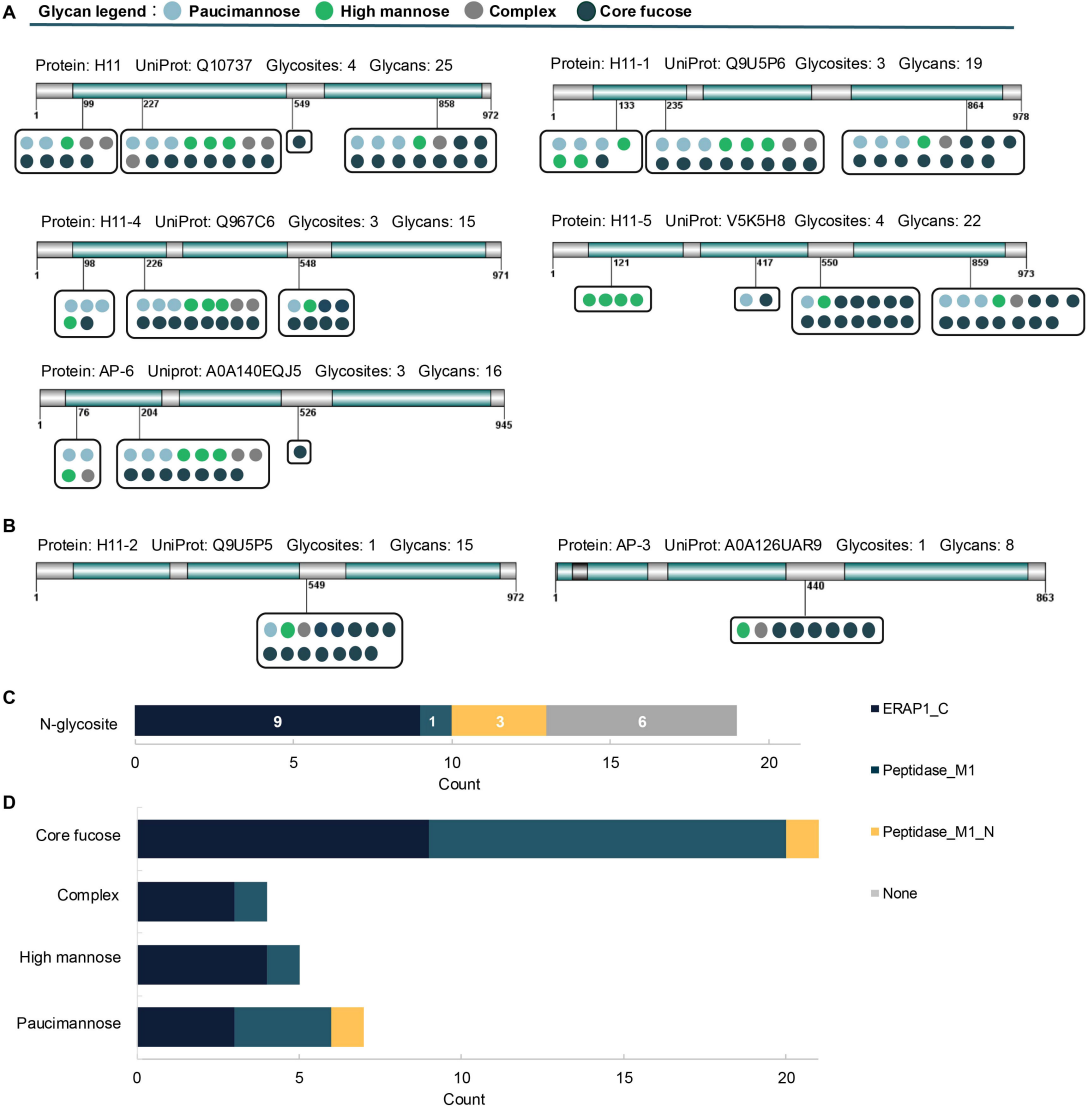
Heterogeneity and protein domain analyses of H11 glycoproteins. **(A, B)** H11 aminopeptidases with one or multiple glycosylation sites, including UniProt accession numbers, number of glycosylation sites, and corresponding glycan structures. **(C)** The number of N-glycosylation sites localized within each domain. **(D)** The number of different glycan types within each protein domain.

Due to the non-template driven nature of glycosylation *in vivo*, natural proteins are typically modified with diverse glycan structures, referring to both macro-heterogeneity (i.e., multiple glycosites within a single protein) and micro-heterogeneity (i.e., multiple glycan structures at individual glycosites) (30). Our heterogeneity analysis of H11 glycoproteins identified 19 N-glycosylation sites with 31 distinct glycoforms (**Figure 2A**), with approximately 89% of glycosylation sites exhibiting micro-heterogeneity. Notably, three hypervariable sites -H11 N^227^, H11–1 N^235^ and H11–4 N^226^-each harbored 16 glycoforms (**Figure 3A**). In terms of macro-heterogeneity, five glycoproteins (H11, H11-1, H11-4, H11-5 and AP-6 contained more than three N-glycosites, whereas H11-2 and AP-3 possessed only a single glycosite (**Figure 3B**). These findings expand upon our previous report of 13 glycosylation sites across six aminopeptidases (15), suggesting that H11 antigen is generally characterized by extensive glycosylation heterogeneity.

To assess the structural context of the glycosylation site on H11 molecules, we analyzed protein domains based on the UniProt database. Our analysis revealed that 13 glycosylation sites were located within three main structural domains: the ERAP1_C domain, the Peptidase_M1 domain, and the Peptidase_M1_N domain (**Figure 3C**). Approximately 47% (n = 9) of the glycosites were assigned to the ERAP1_C domain, with three localized to the Peptidase_M1_N domain and one to the Peptidase_M1 domain (**Figure 3C**). The ERAP1_C domain is reported to form a concave surface associated with aminopeptidase activity, and its arginine–aspartate motif has been implicated as a critical determinant for substrate recognition (31, 32). Comparison of glycan motifs across domains revealed a high proportion of fucosylation in the ERAP1_C and Peptidase_M1 domains (**Figure 3D**). Although functional studies linking protein fucosylation to aminopeptidase activity are currently lacking, this relationship warrants further investigation.

### 3.4 Identification of potential B-cell epitopes within H11 glycopeptide

**Figure 4:**
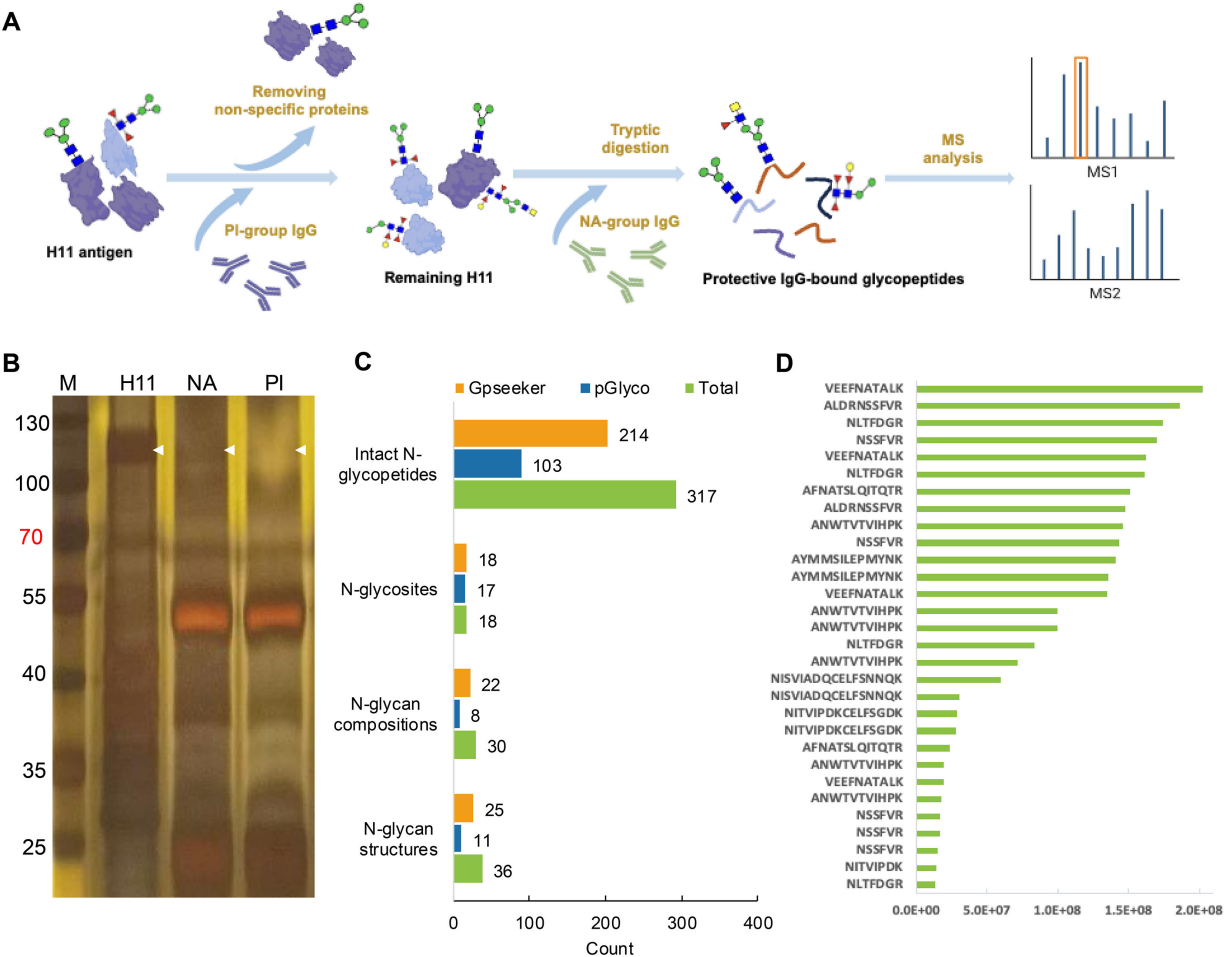
Identification of potential epitopes on H11 glycopeptide. **(A)** Schematic of the experimental procedure for IgG-bound glycopeptide identification. **(B)** Silver staining analysis of native H11 antigen and NA or PI-group IgG-bound H11 antigen. NA-group IgG antibody was induced using natural H11 antigen, and PI-group IgG antibody was made by deglycosylated H11. The white arrow indicates the difference between NA and PI groups in the H11 protein band. **(C)** Numbers of intact N-glycopeptides, N-glycosites, N-glycan compositions, and N-glycan structures from IgG-bound glycopeptides. Data were derived from two independent experiments. **(D)** Enrichment analysis of IgG-bound glycopeptides (Top 30 enriched glycopeptides).

**Figure 5:**
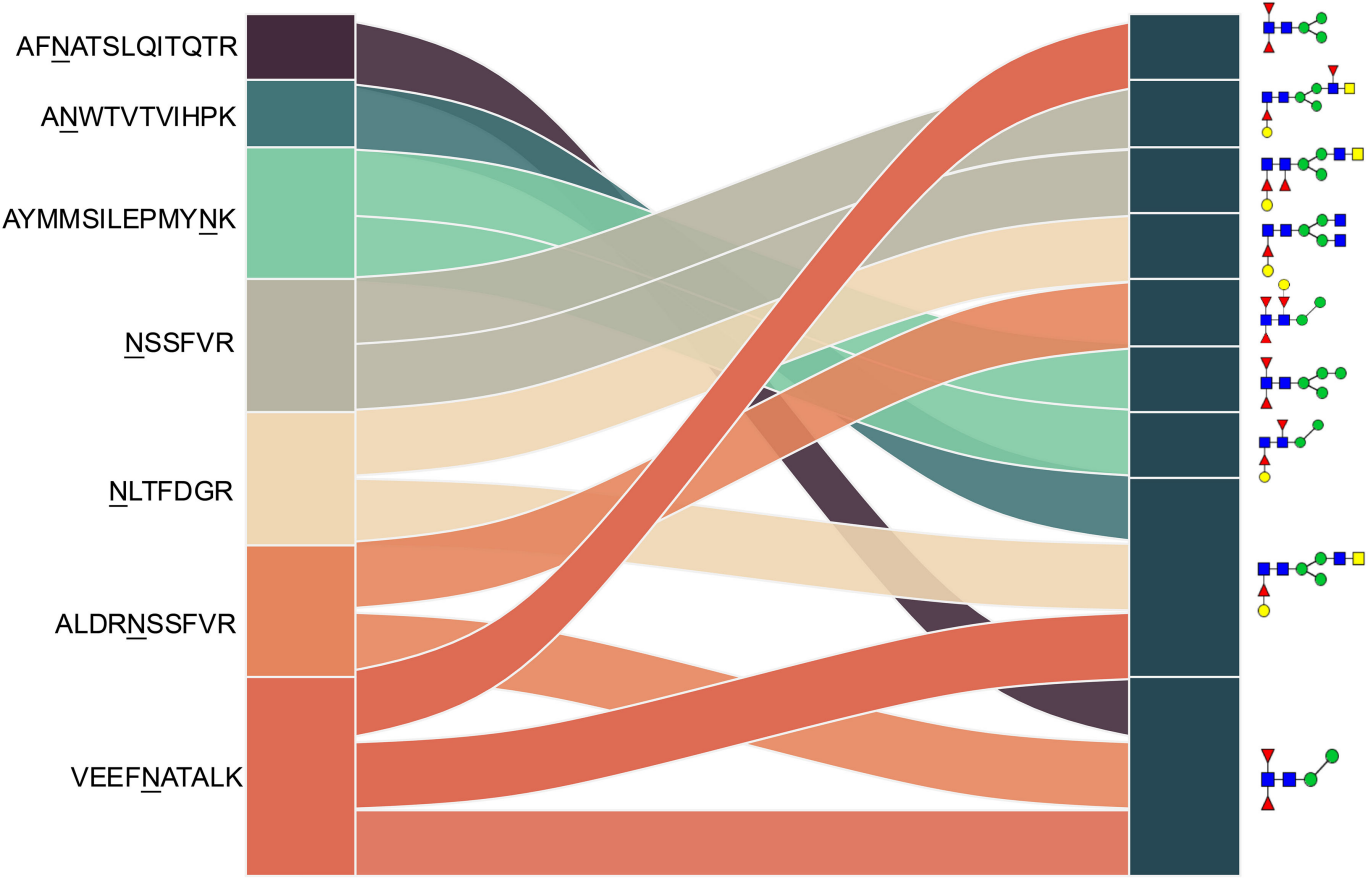
Correlation analysis between H11 glycopeptides and N-glycan structures. Correlation analysis was performed between seven glycopeptides and nine distinct N-glycan structures. Glycans were annotated using the symbol nomenclature (green circle, mannose; yellow circle, galactose; blue square, GlcNAc; yellow square, GalNAc; red triangle, fucose).

Given the importance of N-glycopeptide epitopes, we further explored potential B-cell epitopes recognized by H11-induced IgG antibodies. Silver staining analysis demonstrated that glycosylated H11 was recognized by protective NA-group IgG, but not by PI-group IgG (**Figure 4A, B**). Furthermore, immunoprecipitation combined with glycoproteomic analysis identified 317 intact N-glycopeptides (**Figure 4C**, **Supplementary Table 4**). Among these, 13 glycopeptides exhibited significant enrichment by protective IgG (**Figure 4D**, **Supplementary Table 5**). These glycopeptides were distributed across seven glycosylated aminopeptidases and carried nine distinct N-glycan motifs with core fucosylation. The identified motifs included difucosylated (α1,3- and α1,6-linked) forms, galactosylated α1,6-core fucose, and relatively uncommon trifucosylated structures (**Table 1**). Notably, the VEEFNATALK peptide sequence harbored three distinct N-glycoforms (**Table 1**). We discovered that N-glycan chains of composition Hex_4_HexNAc_4_Fuc_1_ were attached to three peptides (ANWTVTVIHPK, NLTFDGR and VEEFNATALK), whereas Hex_2_HexNAc_2_Fuc_2_ glycan was associated with AFNATSLQITQTR, ALDRNSSFVR and VEEFNATALK (**Figure 5**, **Table 1**). These results highlight the remarkable structural heterogeneity of the potential glycopeptide epitopes present in H11 antigen.

**Table 1:** Mapping of protective IgG-bound intact N-glycopeptides of H11 antigen.

Protein names	N-glycosites	Peptides^a^	Glycans^b^
H11/H11-1/H11-4/ H11-5/AP-3/AP-6	858/864/857/ 859/749/835	NSSFVR	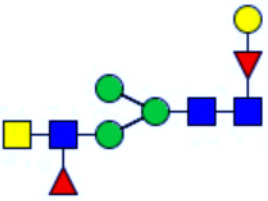
H11/H11-1/H11-4/ H11-5/AP-3/AP-6	858/864/857/ 859/749/835	NSSFVR	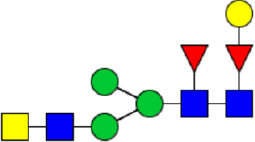
H11/H11-1/H11-5	858/864/859	ALDRNSSFVR	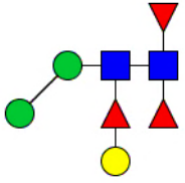
H11/H11-1/H11-5	858/864/859	ALDRNSSFVR	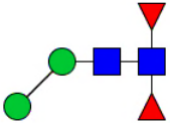
H11/H11-1/H11-4	227/235/226	ANWTVTVIHPK	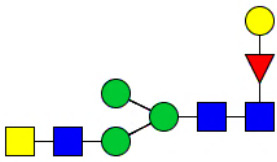
H11/H11-4	99/98	NLTFDGR	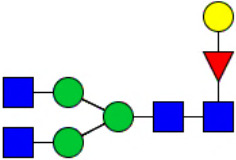
H11/H11-4	99/98	NLTFDGR	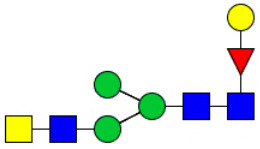
H11-2	549	VEEFNATALK	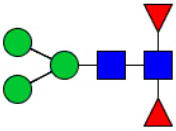
H11-2	549	AYMMSILEPMYNK	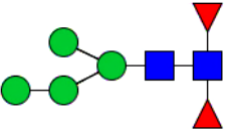
H11-2	549	AYMMSILEPMYNK	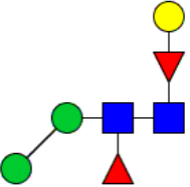
H11-2	549	VEEFNATALK	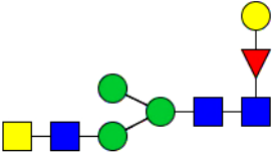
H11-2	549	VEEFNATALK	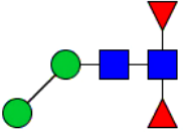
H11-5	550	AFNATSLQITQTR	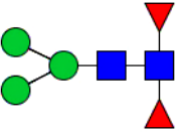

^a^The underlined asparagine (N) indicates the N-glycosylation sites. ^b^Glycans are annotated using the symbol nomenclature (green circle, mannose; yellow circle, galactose; blue square, GlcNAc; yellow square, GalNAc; red triangle, fucose).

**Figure 6:**
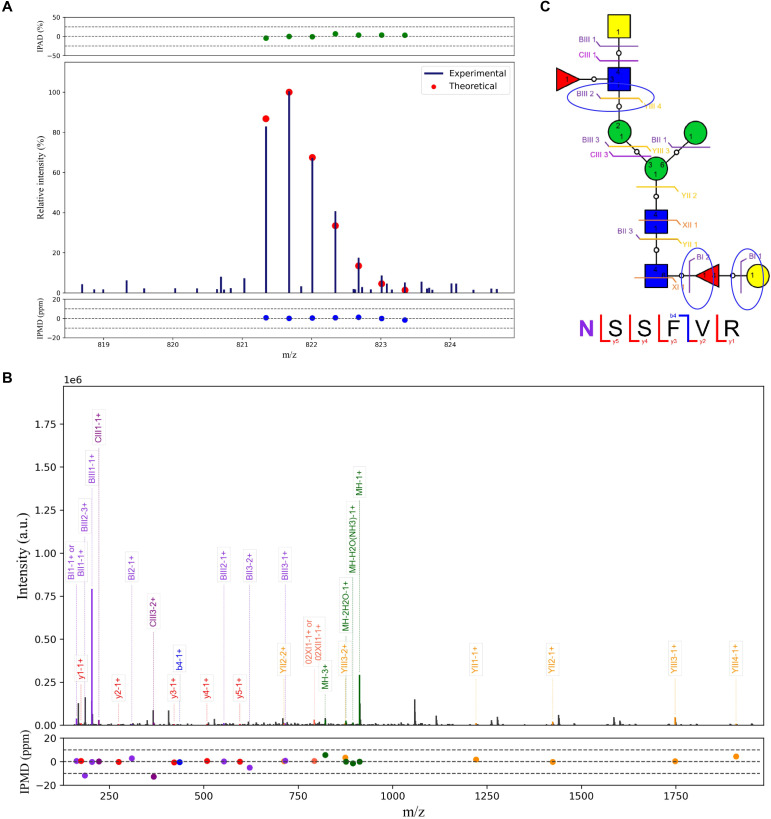
Analysis of potential epitopes on H11 glycopeptide. **(A)** The isotopic envelope finger printing maps of the N-glycopeptide NSSFVR-01Y(61F41L)41Y41M(31M21Y(31F)41V)61M precursor ions. Y: N-acetylglucosamine; M: Mannose; F: Fucose; L: Galactose; V: N-acetylgalactosamine. **(B)** Annotated MS/MS spectrum of matched fragment ions. **(C)** Graphical fragmentations of the peptide backbone and N-glycan structure isomers. Glycans were annotated using the symbol nomenclature (green circle, mannose; yellow circle, galactose; blue square, GlcNAc; yellow square, GalNAc; red triangle, fucose).

In addition, we identified a glycan structure (Hex_3_HexNAc_3_Fuc_2_) attached to the peptide NSSFVR that was specifically recognized by protective IgG. Notably, this glycopeptide was absent from the above H11 antigen analyses, suggesting that it was selectively enriched by protective IgG. MS fragmentation analysis revealed that the BIII2 ion corresponded to a glycan fragment containing two HexNAc residues and one fucose, whereas the BI2 and BI1 ions indicated a hexose linked to a core fucose (**Figures 6A, B**). Database annotation further suggested that this glycan contains a galactosylated α1,6-linked fucose motif with an LDNF (GalNAcβ1,4(Fucα1,3)GlcNAc) unit. Collectively, these findings highlight that N-glycopeptide epitopes represent promising candidates for the development of vaccines targeting *H. contortus* and related parasitic nematodes.

### 3.5 Molecular docking and visual analysis of potential epitopes in H11 glycopeptide

**Figure 7:**
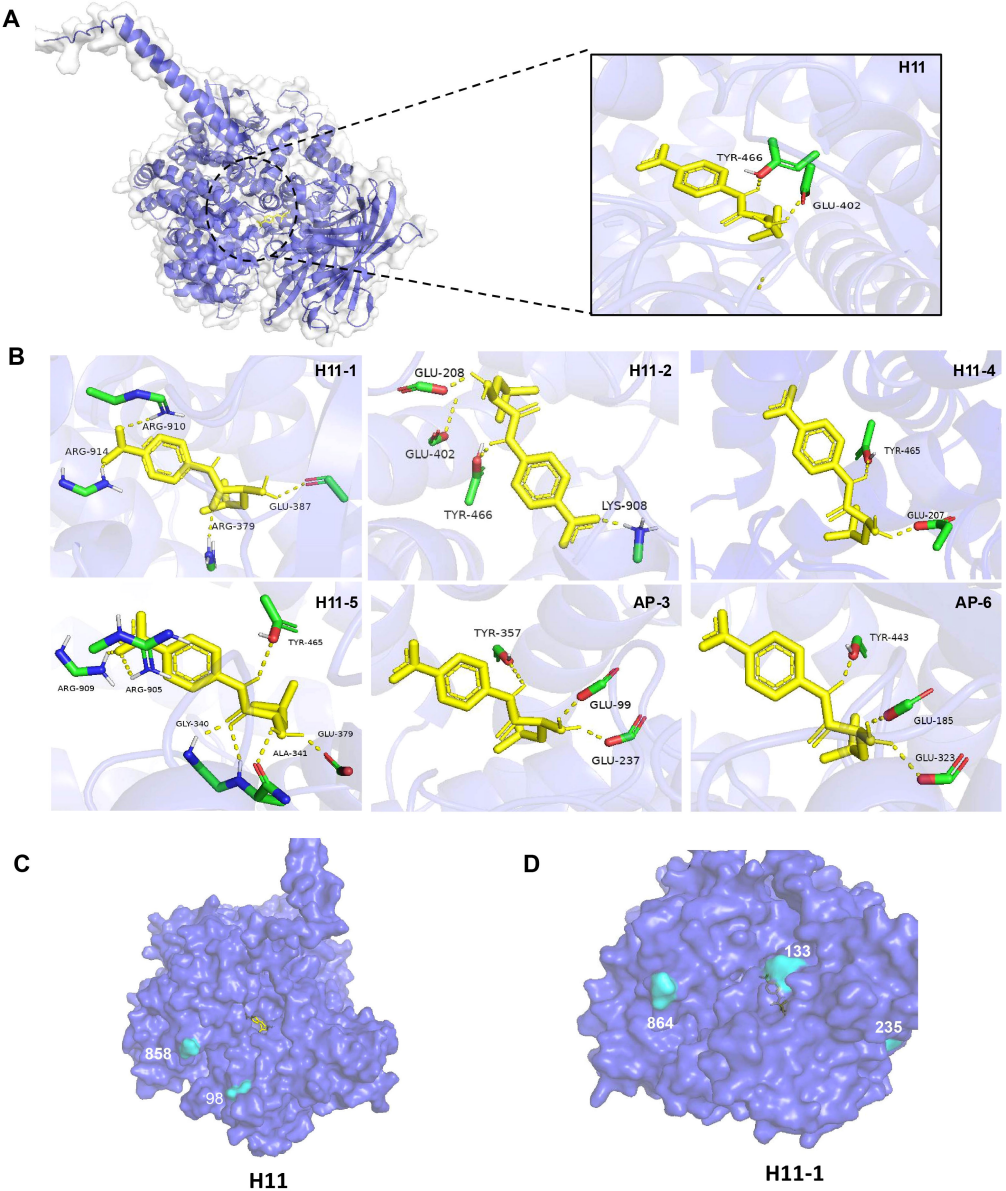
Molecular docking and visualization of identified H11 molecules. **(A)** Complex structure of H11 subtype and substrate (Leu-pNA). **(B)** Local diagram of H11 aminopeptidase amino acid residues interacting with substrate binding. **(C, D)** 3D structures of H11 and H11–2 are shown in Cartoon style.

Protective antibodies can inhibit enzyme activity through steric hindrance or by inducing conformational changes, depending on the spatial relationship between their target epitopes and the enzyme’s active site (33). In this study, we employed Autodock and PyMOL to predict the substrate-binding regions in all identified H11 glycoproteins. Subsequently, three-dimensional structural visualization enabled atomic-level analysis of the interactions between antibodies and their corresponding epitopes. Molecular docking results revealed that the substrate-binding interface is located within the internal cavity of the protein, where the substrate forms multiple hydrogen bonds with key amino acid residues of different H11 isoforms within a 4 Å radius, such as Glu402 and Tyr466 in one H11 protein (**Figures 7A, B**). Structural visualization further allowed us to examine the spatial arrangement between the aminopeptidase active site and antibody-binding epitopes (**Figures 7C, D**, **Supplementary Figure 1**). In the H11 protein, antibody-binding sites were positioned around the substrate-binding channel (**Figure 7C**), whereas in H11-2, these sites were located distally from the catalytic center (**Figure 7D**). Based on these findings, we propose that protective IgG may target those glycopeptide epitopes adjacent to the substrate-binding region, leading to steric blockade or allosteric modulation that interferes with substrate hydrolysis. This mechanism may underlie antibody-mediated inhibition of aminopeptidase activity and contribute to protective immunity. Further characterization of these glycopeptide epitopes will be instrumental in guiding the rational design of anthelmintic drugs and vaccines against *H. contortus*.

## 4 Discussion

H11 antigen is a well-characterized immunoprotective molecule from a parasitic nematode. In its native form, H11 consistently confers high levels of protection, typically ranging from 75% to 95% in immunized animals (17). In spite of substantial academic–industry efforts, recombinant H11 proteins produced in both prokaryotic and eukaryotic systems have failed to elicit comparable levels of protective immunity in animal models (22, 34). To elucidate the mechanisms underpinning its potent immunogenicity, our previous work characterized the glycome and glycoproteome of the native H11 antigen, emphasizing the critical contribution of glycan structures (15). However, a detailed site- and structure-specific glycoproteins profile of H11 remained unknown.

Here we performed the first comprehensive N-glycopeptides analysis of *H. contortus* H11 antigen. Using MS-based intact glycoproteomics and integrating GPSeeker and pGlyco platforms, our analysis identified seven of the 13 aminopeptidases, including five H11 isoforms (H11, H11–1, H11-2, H11–4, and H11-5) and two additional aminopeptidase molecules (AP-3 and AP-6). Among them, five known H11 isoforms, including H11-1, H11-2, H11-4, and H11-5 have been reported to be associated with immunogenicity in previous studies (11, 18). Although the immunogenicity of two additional aminopeptidase molecules (AP-3 and AP-6) has not yet been verified, their potential immune functions deserve further investigation. Correlation analysis of glycosylation sites and glycan motifs showed that these seven molecules harbored 19 glycosylation sites with 31 glycan structures, showing a high degree of heterogeneity among H11 glycoproteins. Although our previous study revealed the N-glycoproteome and glycan profiles (15), it still lacked information linking specific glycan chains to their corresponding peptides. So far, decoding the intact glycoproteome of parasitic worms remains a significant technical challenge due to the structural complexity and species-specific nature of their glycans. Helminth glycans are often characterized by highly branched architectures, non-mammalian monosaccharide residues, and extensive fucosylation (35, 36), all of which hinder conventional analytical workflows. Despite these challenges, site-specific glycosylation analysis is essential, as intact glycoproteins play crucial roles in parasite biology, immune recognition, and vaccine efficacy (37, 38).

We demonstrated that 13 N-glycopeptides were selectively enriched by protective IgG antibodies. Detailed characterization revealed a high degree of structural heterogeneity and extensive core fucosylation, encompassing difucosylated, galactosylated α1,6-linked monofucosylated, and rare trifucosylated motifs. Core fucose motifs have been identified in previous glycomic studies of *H. contortus* (39, 40). Notably, the immunomodulatory roles of core fucosylation have been highlighted in *H. contortus* and other parasitic helminths. For instance, α1,3-core fucose present on *H. contortus* excretory/secretory glycoproteins functions as a B-cell epitope, as evidenced by its recognition by IgG antibodies from vaccinated animals (41). In addition, α1,3-core fucose has been specifically identified on glycoprotein omega-1 from *Schistosoma mansoni* eggs and is associated with its Th2-inducing property (42). Moreover, LDNF epitopes have been shown to be targeted by IgG antibodies in sheep immunized with *H. contortus* excretory/secretory glycoproteins (43). Similar glycan motifs have also been exploited as immunogenic glycans in the diagnosis and development of glycoconjugate vaccines against *S. mansoni* (44, 45).

Molecular docking studies demonstrated that the IgG-recognized N-glycopeptides are located on the protein surface or near the substrate entry channels leading to the aminopeptidase active sites. This spatial localization suggests a potential immunological mechanism in which antibody binding sterically hinders substrate access or enzyme activity, thereby disrupting key physiological functions of the parasite aminopeptidases. Our previous work has demonstrated that protective IgG antibodies targeted glycan structures to inhibit enzymatic activity, and this inhibition is closely associated with the development of immunoprotection (15). Furthermore, the surface exposure of these glycopeptide epitopes increases their accessibility to host immune surveillance, thereby enhancing their relevance as immunodominant targets. These findings support the hypothesis that the spatial positioning of glycopeptide epitopes is a key determinant of protective immunity and emphasize the necessity of preserving native glycosylation patterns in the development of effective subunit vaccines. However, while molecular docking offers valuable insights into the surface localization of IgG-recognized N-glycopeptides, it cannot fully capture the complexity of the attached glycan structures, limiting our ability to assess how specific glycan modifications affect antibody binding and enzymatic inhibition.

Substantial studies have shown that N-glycopeptides can possess an effective immunogenicity (46). For example, HIV-neutralizing antibodies targeting conserved gp120 glycopeptides have successfully elicited robust immune responses in animal models (47, 48). Despite substantial progress in helminth vaccine research, glycopeptide epitopes relevant to protective immunity have yet to be clearly defined in parasitic worms. Previous studies have focused primarily on either glycan or protein components in isolation (15, 39, 40), lacking the site-specific linkage information. Such information is essential for elucidating how specific glycan structures contribute to antigen recognition and immune protection. A comprehensive understanding of these H11 glycoproteins will therefore offer new molecular insights into the mechanisms of protective immunity and guide the rational design of effective glycoengineered subunit vaccines. Recent studies have successfully expressed glycoengineered H11 antigens in insect cells (49, 50). Glycoengineering has been shown to offer precise control of antigen composition, enhance safety, and allow scalable production, thereby addressing major challenges in vaccine development (51). Collectively, these findings highlight the indispensable role of native glycosylation patterns and epitope positioning in determining vaccine efficacy, paving the way for the rational development of next-generation antihelminthic vaccines.

## Data Availability

The original contributions presented in the study are publicly available. This data can be found here: https://www.ebi.ac.uk/pride/archive/; PXD number PXD068910.

## Supplementary material

Supplementary Figure 1 Molecular docking and visualization of identified H11 molecules. 3D structures of H11-1 **(A)**, H11-4 **(B)**, H11-5 **(C)**, AP-3 **(D)**, and AP-6 **(E)** are shown in Cartoon styles.
