# Supplementary figures and images for: Site- and structure-specific characterization of glycoproteins of H11: potent vaccine candidates against parasitic worm Haemonchus
Source: Front Immunol. 2026 Jan 5;16:1669536. doi: 10.3389/fimmu.2025.1669536 (PMC12813067; doi:10.3389/fimmu.2025.1669536)

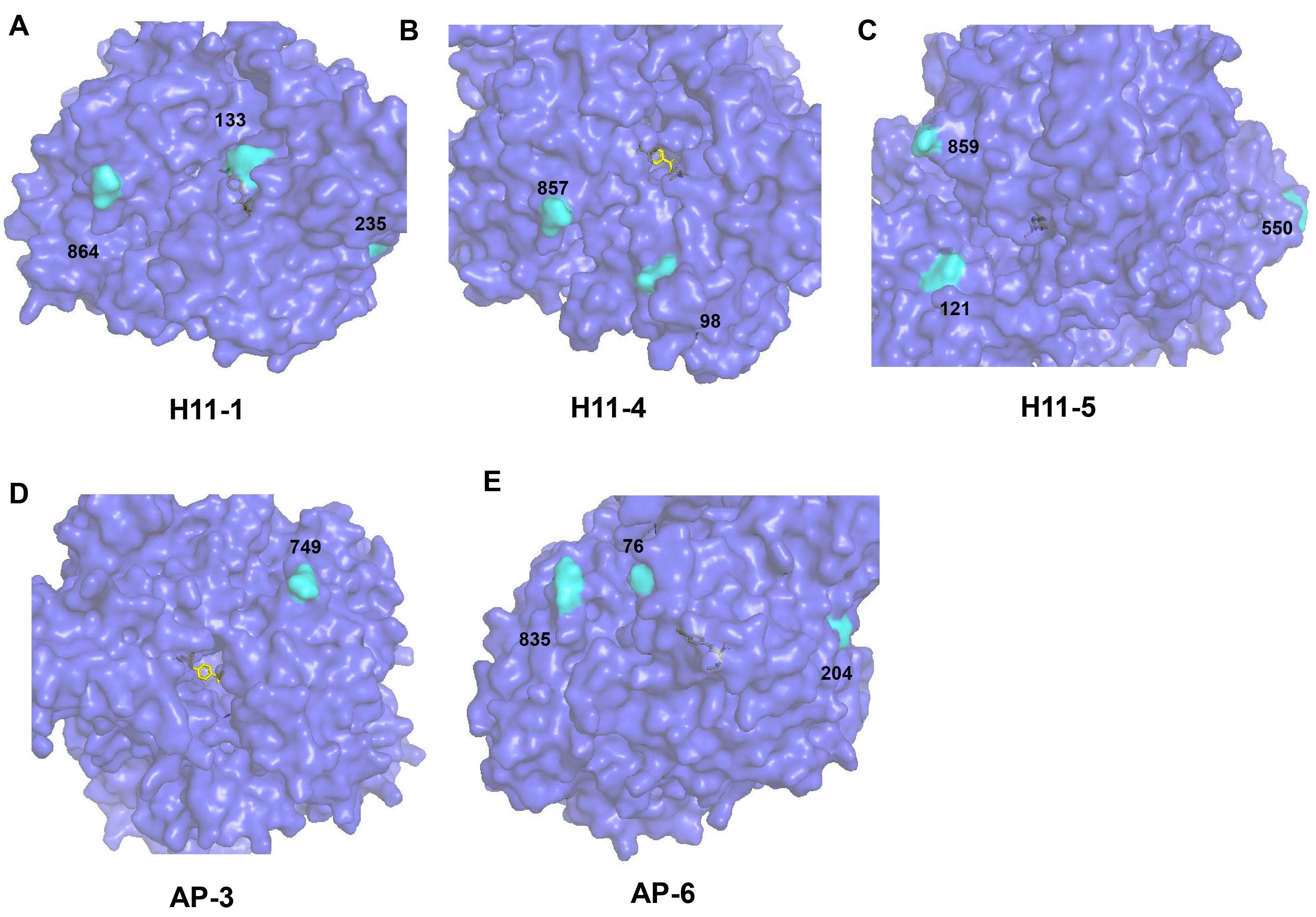

Supplement: Supplementary Figure 1 — Molecular docking and visualization of identified H11 molecules. 3D structures of H11-1 (A), H11-4 (B), H11-5 (C), AP-3 (D), and AP-6 (E) are shown in Cartoon styles. [file Image1.jpeg]
